# ABC transporter genes *ABC-C6* and *ABC-G33* alter plant-microbe-parasite interactions in the rhizosphere

**DOI:** 10.1038/s41598-019-56493-w

**Published:** 2019-12-27

**Authors:** Deborah Elizabeth Cox, Steven Dyer, Ryan Weir, Xavier Cheseto, Matthew Sturrock, Danny Coyne, Baldwyn Torto, Aaron G. Maule, Johnathan J. Dalzell

**Affiliations:** 1 School of Biological Sciences, Institute for Global Food Security, Queen’s University Belfast, Belfast, UK; 2 The International Center of Insect Physiology and Ecology, Nairobi, Kenya; 3 The International Institute for Tropical Agriculture, Nairobi, Kenya

**Keywords:** Plant signalling, Animal behaviour

## Abstract

Plants are master regulators of rhizosphere ecology, secreting a complex mixture of compounds into the soil, collectively termed plant root exudate. Root exudate composition is highly dynamic and functional, mediating economically important interactions between plants and a wide range of soil organisms. Currently we know very little about the molecular basis of root exudate composition, which is a key hurdle to functional exploitation of root exudates for crop improvement. Root expressed transporters modulate exudate composition and could be manipulated to develop beneficial plant root exudate traits. Using Virus Induced Gene silencing (VIGS), we demonstrate that knockdown of two root-expressed ABC transporter genes in tomato cv. Moneymaker, *ABC-C6* and *ABC-G33*, alters the composition of semi-volatile compounds in collected root exudates. Root exudate chemotaxis assays demonstrate that knockdown of each transporter gene triggers the repulsion of economically relevant *Meloidogyne* and *Globodera* spp. plant parasitic nematodes, which are attracted to control treatment root exudates. Knockdown of *ABC-C6* inhibits egg hatching of *Meloidogyne* and *Globodera* spp., relative to controls. Knockdown of *ABC-G33* has no impact on egg hatching of *Meloidogyne* spp. but has a substantial inhibitory impact on egg hatching of *G. pallida. ABC-C6* knockdown has no impact on the attraction of the plant pathogen *Agrobacterium tumefaciens*, or the plant growth promoting *Bacillus subtilis*, relative to controls. Silencing *ABC-G33* induces a statistically significant reduction in attraction of *B. subtilis*, with no impact on attraction of *A. tumefaciens*. By inoculating selected differentially exuded compounds into control root exudates, we demonstrate that hexadecaonic acid and pentadecane are biologically relevant parasite repellents. *ABC-C6* represents a promising target for breeding or biotechnology intervention strategies as gene knockdown leads to the repulsion of economically important plant parasites and retains attraction of the beneficial rhizobacterium *B. subtilis*. This study exposes the link between ABC transporters, root exudate composition, and *ex planta* interactions with agriculturally and economically relevant rhizosphere organisms, paving the way for new approaches to rhizosphere engineering and crop protection.

## Introduction

Plants secrete a complex mixture of water soluble and volatile organic compounds (VOCs) into the soil, collectively termed plant root exudate. Root exudates can enhance the recruitment of beneficial microbes^1–5^, mobilise nutrients^6^, sequester toxic compounds in the soil^7^, and communicate with other plants, and animals^8,9^. Root exudate composition is dynamic and can be modulated as a factor of development^10,11^, environment^12^, physiological state^5^, and displays marked diversity between species^13–16^ and cultivars^17–21^. It is estimated that between 5% and 21% of all photosynthetically-assimilated carbon is released as root exudate^22,23^.

The process of crop domestication has focused on a small number of desirable traits, relating to plant stature, yield and disease resistance^24^ often at the expense of other traits. There is evidence that the domestication process has exerted a significant and unintended impact on root exudate composition, and rhizosphere microbe interactions^25,26^. A general lack of understanding and mechanistic insight represents a major impediment to the exploitation of root exudates for crop improvement. However, it is clear that root exudate composition is an adaptive trait, which can be manipulated. For example, much progress has been made in understanding the interaction between root exudates, and parasitic *Striga* spp. over recent years. This insight has underpinned efforts to alter exudate strigolactone content, a known germination stimulant and attractant of these economically important and globally distributed parasitic plants^17,27–30^. Comparatively, less progress has been made in understanding the analogous interaction between root exudate and plant parasitic nematodes (PPNs), in part due to the increased complexity of nematode biology. Nonetheless, recent years have seen renewed interest in this field of research^18,31–35^.

It is estimated that PPNs reduce crop yields by 12.3%, equating to an estimated $US 80–157 billion in losses each year^36–38^. PPNs respond transcriptionally, physiologically and behaviourally to plant root exudates, using exudates to trigger egg hatching, and to facilitate host-finding^31,35,39–43^. Understanding the molecular, chemical and physiological mechanisms underpinning both root exudation and PPN interactions could facilitate the development of aggressive new *ex planta* control strategies for sustainable intensification of global agriculture, through breeding, rhizosphere engineering and/or biotechnology^44–47^. The identification of key parasite attractants and repellents could also facilitate the development of new push-pull strategies.

The rhizosphere microbiome has a major influence on crop health^48,49^, and phenotype^50,51^. Microbial chemotaxis to plant root exudates is an important factor in the competition for chemical resources in the rhizosphere, and colonisation of plant roots^3,52^. As such, alteration of root exudate composition could impact on a wide range of interactions. Exploitation of root exudates for improved crop health offers intriguing potential, but requires a detailed study of the link between crop genotype and highly complex, multi-species interactions.

Considerable interest has developed around the manipulation of membrane transporters for crop improvement^53,54^, and ABC transporters have been implicated directly in modifying root exudate composition^5,55^. ABC transporters represent one of the single largest gene families in plants, which regulate the sequestration and mobilisation of a vast array of chemistry linked to diverse metabolic, physiological and morphological functions^56–60^. The ABC transporter gene complement of tomato is numbered at 154, with a considerable proportion expressed in root tissue^61^. Here we have employed an improved Virus Induced Gene Silencing (VIGS) method to reveal a functional link between two ABC transporter genes, root exudate composition, and rhizosphere interactions with economically important microbes and parasites.

## Results

### Knockdown of tomato ABC transporter genes by VIGS

VIGS was used to co-silence two genes of interest (*ABC-C6* or *ABC-G33*), alongside a visual reporter gene, Phytoene DeSaturase (*PDS*). Knockdown of *PDS* triggers a mild leaf bleaching phenotype due to the accumulation of phytoene, a white carotenoid pigment^62^. Co-silencing was necessary to identify responsive plants for exudate collection and downstream bioassays; not all plants will trigger a viable RNAi response to VIGS challenge. We observed that plant growth was reduced by over 75% relative to control treatments when using the traditional blunt syringe inoculation method. We therefore sought to develop a less damaging approach to inoculation of *A. tumefaciens* (containing the VIGS plasmids). We discovered that *A. tumefaciens* cultures could efficiently invade leaf cells when applied topically to tomato seedling cotyledons with Silwett L-77, which is frequently used to aid *A. tumefaciens* invasion during floral dip transformation protocols^63^. Following topical application of *A. tumefaciens* (containing the VIGS plasmids), bleaching phenotypes typically began to develop after 11 days and peaked at around 21 days post inoculation. Bleaching phenotypes following blunt syringe application began to emerge around 15 days post inoculation, and similarly peaked around 21 days post inoculation. Furthermore, the frequency of plants demonstrating the mild photobleaching phenotype associated with *PDS* knockdown was between 80% and 100% following topical application. The leaf infiltration method resulted in around 75% of plants demonstrating the co-silenced bleaching phenotype.

**Figure 1:**
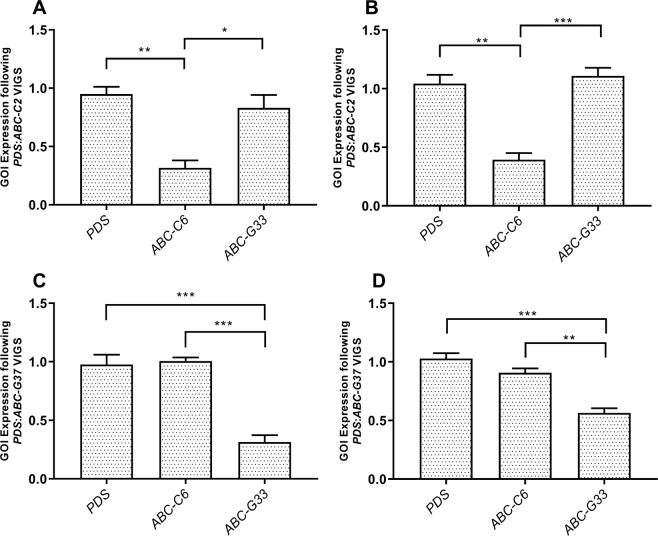
VIGS triggers target-specific knockdown of root-expressed ABC transporter genes in tomato. (**A**) The mean ratio of *ABC-C6* abundance relative to the endogenous control gene, elongation factor 1 subunit alpha (*EF-α*), following blunt syringe inoculation of *A. tumefaciens* and pTRV plasmids. (**B**) The mean ratio of *ABC-C6* abundance relative to the endogenous control gene following topical application of *A. tumefaciens* and pTRV plasmids. (**C**) The mean ratio of *ABC-G33* abundance relative to the endogenous control gene following blunt syringe inoculation of *A. tumefaciens* and pTRV plasmids. (**D**) The mean ratio of *ABC-G33* abundance relative to the endogenous control gene following topical application of *A. tumefaciens* and pTRV plasmids. Data represent three biological replicates sampled on day 21 post-inoculation, with each replicate consisting of three plants each; error bars represent SEM. One-way ANOVA and Tukey’s HSD tests were used to assess statistical significance between groups (*P < 0.05, **P < 0.01, ***P < 0.001).

Both inoculation methods triggered robust and specific gene knockdown by week three post inoculation (Fig. 1), with some gene-specific variation around the relative persistence of gene knockdown in topical application treatment groups, relative to syringe inoculation treatment groups. Plants were sampled three weeks post inoculation for further experiments. Transcript abundance of *ABC-C6* and *ABC-G33* was reduced by 63.29% ± 11.64% and 66.30% ± 8.68% using blunt syringe inoculation. Topical application resulted in transcript knockdown of 64.92% ± 9.44% and 46.40% ± 5.90% for *ABC-C6* and *ABC-G33* respectively. *ABC-C6* transcript levels recovered to control treatment levels by week nine post inoculation when syringe inoculation was employed. In contrast, *ABC-G33* transcript abundance remained significantly reduced at week nine following topical inoculation. Due to the improved performance of topical inoculation in terms of knockdown persistence and plant growth rate, we adopted this method for all subsequent experiments. Only plants that displayed co-silenced bleaching phenotypes were taken for further analysis across all experiments.

### Knockdown of ABC transporter genes modulate exudate composition and parasite behaviour

Root exudate was collected from VIGS treatment groups at three weeks post inoculation. Behavioural responses of two root knot nematodes, *Meloiodogyne incognita* and *Meloidogyne javanica*, alongside the potato cyst nematode, *Globodera pallida* were assayed across experimental exudates.

### Hatching

**Figure 2:**
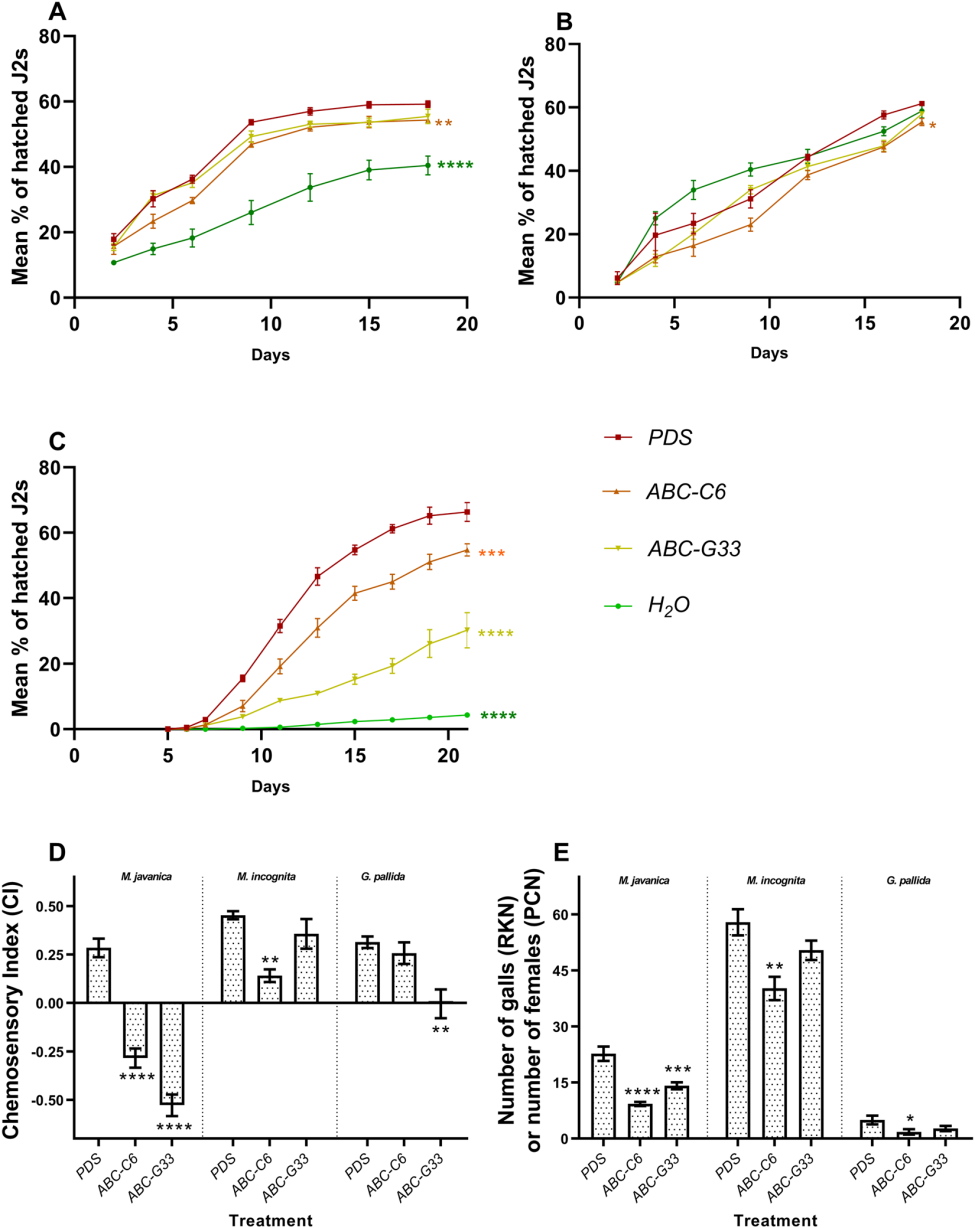
ABC transporter gene knockdown modulates PPN hatching, attraction and invasion. Hatching of J2s in response to experimental treatment (root exudates or water) as a factor of time, for (**A**) *M. incognita*; (**B**) *M. javanica*; and (**C**) *G. pallida*. Error bars represent SEM. (**D**) Chemosensory challenge of *Meloidogyne* and *Globodera* spp. A positive chemosensory index (CI) equates to attraction towards tested exudates, and a negative CI infers repulsion from the exudates. Data represent five biological replicates for each nematode species in each treatment group. (**E**) The number of galls formed (*Meloidogyne* spp.) or developing females found on the root surface (*G. pallida*) six weeks post inoculation on VIGS plants (n = 10 plants per species). Asterisks indicate statistical significance relative to controls following one-way ANOVA and Tukey’s HSD tests: *P < 0.05; **P < 0.01; ***P < 0.001; ****P < 0.0001.

The hatching response of each species was measured as the percentage of emerging infective second stage juveniles (J2s) over time, for which the area under the curve was calculated for comparison of experimental groups (Fig. 2A–C). Area under the curve allows a more robust assessment of hatching phenotypes over time, as it is proportional to both the rate of hatching (gradient) and also the final hatch percentage. Knockdown of *ABC-C6* and *ABC-G33* triggered a reduction in *G. pallida* hatching (measured as area under the cumulative percentage hatch [AUCPH]) following knockdown of *ABC-C6* and *ABC-G33* by 174.40 ± 22.04 (arbitrary units) and 420.10 ± 23.29, respectively (relative to *PDS* control). Egg hatching was also reduced for *M. incognita* following knockdown of *ABC-C6*, by 33.80 ± 7.31. Knockdown of *ABC-G33* caused hatching reduction of 16.90 ± 7.11. Knockdown of *ABC-C6* also triggered a reduction in hatching of *M. javanica* of 112.40 ± 33.31, whereas knockdown of *ABC-G33* led to a reduction of 59.70 ± 30.83.

### Attraction

Knockdown of *ABC-C6* modified the chemosensory responses of *M. incognita* and *M. javanica* to collected exudates (Fig. 2D). Experimentally manipulated exudates were less attractive to *M. incognita* J2s, whereas *M. javanica* J2s were repelled by the same exudates. Following knockdown of *ABC-C6*, chemosensory indices (CI) were reduced by 0.31 ± 0.04 and 0.57 ± 0.07 for *M. incognita* and *M. javanica* respectively, relative to *PDS* control exudates. Following knockdown of *ABC-G33*, *M. javanica* was consistently repelled by these exudates with a CI score reduction of 0.81 ± 0.07, whereas *M. incognita* retained attraction. The CI score for *M. incognita* to *ABC-G33* exudates was reduced by just 0.10 ± 0.08. *G. pallida* J2s displayed reduced attraction to exudates from *ABC-G33* knockdown plants, by 0.32 ± 0.08, whereas for *ABC-C6* exudates, the CI was reduced by only 0.06 ± 0.06.

### Plant infection

Knockdown of ABC transporter genes also reduced the number of galls produced following infection of VIGS plants with *Meloidogyne* spp. (Fig. 2E); knockdown of *ABC-C6* and *ABC-G33* caused a reduction of 17.70 ± 4.68 galls and 7.50 ± 4.34 galls respectively, following infection with *M. incognita*. The number of galls produced following infection by *M. javanica* was reduced by 13.43 ± 2.02 and 8.57 ± 2.14 when *ABC-C6* and *ABC-G33* knockdown plants were challenged, respectively. The number of *G. pallida* females was reduced by 3.20 ± 1.38 following knockdown of *ABC-C6*, whereas knockdown of *ABC-G33* caused a decrease of 2.30 ± 1.36 cysts per plant. *G. pallida* retained attraction to exudates collected following *ABC-C6* knockdown, however cyst counts were significantly reduced following infection of VIGS plants.

### Metabolomic characterisation of exudates following ABC transporter knockdown

**Figure 3:**
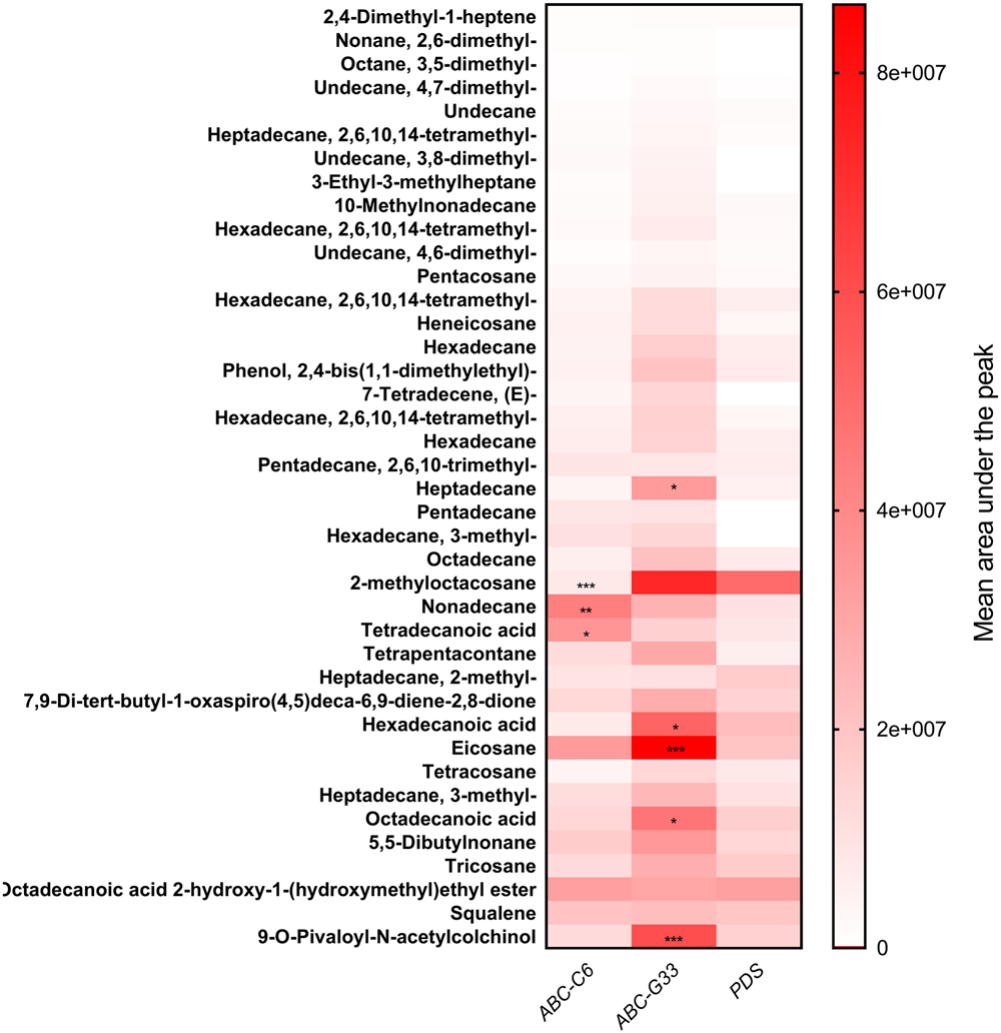
Heatmap showing differences in the relative abundance of identified compounds across experimental exudates. The mean composition of 10 biological replicates (three plants per replicate) is plotted for each experimental group post-VIGS, and has been assessed by two-way ANOVA, and Tukey’s multiple comparison test. Statistical significance is indicated relative to the *PDS* knockdown control, *P < 0.05, **P < 0.01, ***P < 0.001.

Collected exudates were assessed by coupled gas chromatography-mass spectrometry (GC-MS) to identify changes in exudate composition. Several compounds were quantitatively altered in exudates collected following ABC gene knockdown, relative to *PDS* knockdown controls (Fig. 3). Knockdown of *ABC-C6* resulted in reduced abundance of 2-methyloctacosane, and increased abundance of nonadecane and tetradecanoic acid, relative to control treatment (*PDS* knockdown). Contrastingly, knockdown of *ABC-G33* triggered elevated abundance of eicosane, 9-O-pivaloyl-N-acetylcolchinol, heptadecane and octadecanoic acid. Exudate compounds were quantified across experimental groups, and fatty acid composition was confirmed by derivatization using their methyl esters (Supplementary File S1).

### Parasite behavioural responses to selected differentially exuded compounds

**Figure 4:**
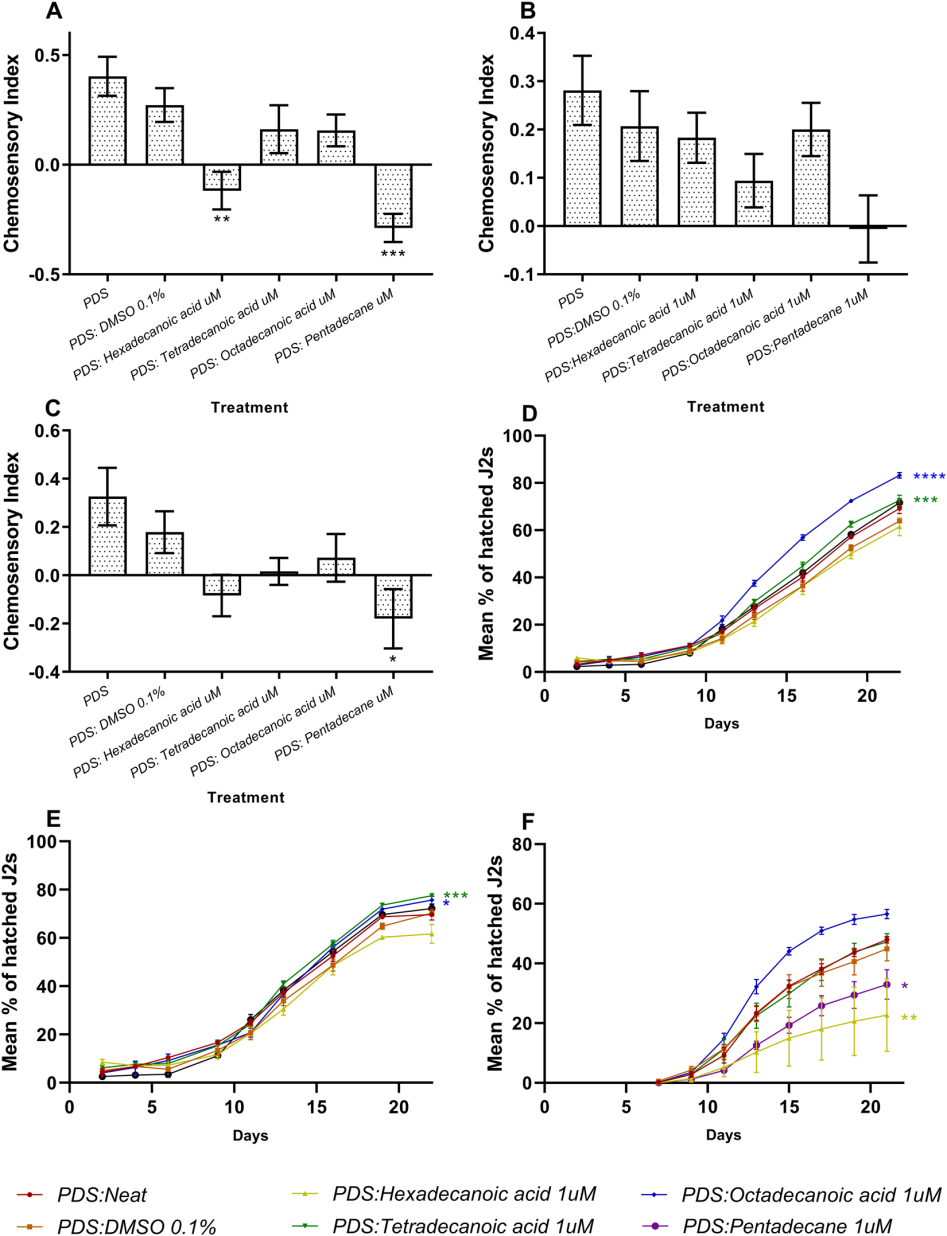
PPN responses to selected exudate compounds. Chemotaxis responses to selected compounds inoculated into control plant root exudate (*PDS* knockdown treatment) for: (**A**) *M. incognita*; (**B**) *M. javanica*; (C) *G. pallida*. Data represent the mean of five biological replicates of root exudate, for which ten replicate assays are performed for each nematode species. Asterisks indicate statistical significance in chemosensory index relative to the PDS:DMSO control following one-way ANOVA and Tukey’s HSD tests. Hatch responses following inoculation of control exudate with selected compounds for: (**D**) *M. incognita*; (**E**) *M. javanica*; (**F**) *G. pallida*. The area under the curve of percentage hatch (AUCPH) was estimated by trapezoidal integration and compared by one-way ANOVA and Dunnett’s multiple comparisons test and asterisks indicate statistical significance in hatching relative to the *PDS*:DMSO control. **P < 0.01; ***P < 0.001; ****P < 0.0001, error bars represent SEM.

PPN species were assayed for responsiveness to selected commercially available compounds that were differentially exuded following knockdown of either ABC transporter gene. Tetradecanoic acid (elevated following *ABC-C6* knockdown), hexadecanoic acid, octadecanoic acid (both elevated following *ABC-G33* knockdown), and pentadecane (elevated following knockdown of both *ABC-C6* and *ABC-G33* relative to controls) were solubilised in 100% dimethyl sulfoxide (DMSO) (1 mM stocks). Each solubilised compound was independently inoculated into control exudates (*PDS* knockdown) to a final experimental concentration of 1 µM 0.10% DMSO. Inoculated control exudates were then used for egg hatching and chemotaxis assays (Fig. 4).

Egg hatching of *Meloidogyne* spp. was enhanced when tetradecanoic acid or octadecanoic acid were inoculated into *PDS* knockdown exudates. The AUCPH for *M. incognita* increased by 95 ± 16.82% days and 208.10 ± 17.22% days, respectively (Fig. 4A). For *M. javanica* the AUCPH increased by 101.70 ± 19.52 and 71.20 ± 21.76, respectively (Fig. 4B). However, the addition of hexadecanoic acid and pentadecane significantly inhibited egg hatching in *G. pallida* by 178.70 ± 65.22% days and 124.20 ± 31.80% days, respectively (Fig. 4C).

The addition of 1 µM pentadecane to control root exudates reduced the CI of *M. incognita* by 0.56 ± 0.10. Likewise, the addition of 1 µM hexadecanoic acid reduced the CI of *M. incognita* by 0.39 ± 0.12. 1 µM tetradecanoic acid, or octadecanoic acid had no statistically significant impact on *M. incognita* attraction to control root exudates. For *M. javanica*, a 0.21 ± 0.10 decrease in CI was observed upon addition of 1 µM pentadecane; no statistically significant differences were observed following addition of the other compounds. The CI of *G. pallida* to root exudates was reduced by 0.36 ± 0.15 with the addition of 1 µM pentadecane.

### Knockdown of ABC transporter genes selectively modulates microbial chemotaxis

**Figure 5:**
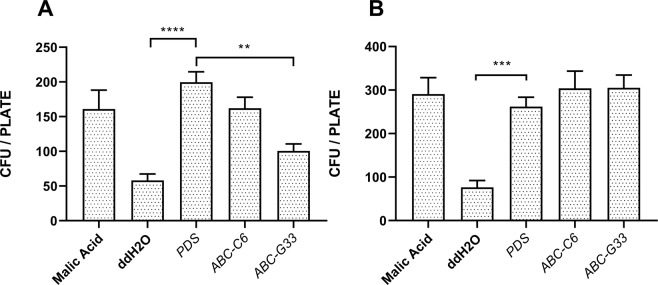
ABC transporter gene knockdown modulates the attraction of *B. subtilis*, but not *A. tumefaciens*. (**A**) Chemosensory response of *B. subtilis* (168) to root exudates collected following gene knockdown. (**B**) Chemosensory response of *A. tumefaciens* (AGL-1) to root exudates collected following gene knockdown. Data represent ten biological replicates for each species. **P < 0.01; ***P < 0.001; ****P < 0.0001, error bars represent SEM.

The attraction of *B. subtilis* and *A. tumefaciens* to root exudates was assessed following ABC gene knockdown. Both species were significantly more attracted to the positive control, 1 mM malic acid, relative to the negative ddH_2_O control. Similarly, root exudates collected from the *PDS* knockdown plants were significantly more attractive to both *B. subtilis* and *A. tumefaciens*, than ddH_2_O (Fig. 5). The attraction of *B. subtilis* to exudates collected following knockdown of *ABC-C6* was statistically unchanged relative to the control treatment. However, knockdown of *ABC-G33* triggered a reduced attraction, and a mean reduction of 99 colony forming units (CFUs) relative to exudates from *PDS* knockdown plants. Knockdown of *ABC-C6* and *ABC-G33* had no impact on the attraction of *A. tumefaciens* to collected exudates.

## Discussion

The predominantly sessile life-style of a plant necessitates substantial molecular and biochemical plasticity to coordinate responses to environmental conditions, and to interact contextually with floral and faunal communities. It is clear that plant genotype influences exudate composition, and organismal interactions^17–21,25,26^. However, our understanding of the basic biology underpinning these interactions is limited.

ABC transporters modulate root exudate composition, and rhizosphere microbe interactions^5,48,55^. In this study, we used VIGS to investigate the role of two ABC transporter genes, *ABC-C6* and *ABC-G33*, in modulating tomato root exudate composition, and interactions with three important PPN species. Specifically, we demonstrate that knockdown of *ABC-C6* and *ABC-G33* transporter genes quantitatively alters tomato root exudate composition and inhibits PPN hatching and attraction behaviours to varying degrees. We assessed the involvement of individual compounds in mediating interactions with the PPN species by inoculating selected, differentially exuded compounds into control exudates. By assessing hatching and chemotaxis responses to these experimentally manipulated exudates, we demonstrate that hexadecanoic acid and pentadecane are inhibitors of *G. pallida* host-finding and hatching; pentadecane is an inhibitor of *Meloidogyne* spp. host-finding. Tetradecanoic acid and octadecanoic acid had no impact on host-finding of either species but did enhance egg hatching rates of both *Meloidogyne* spp. These results suggest that hydrocarbons and fatty acids in root exudate both mediate PPN host finding. However, our findings do not exclude the possibility that other classes of compounds may play a role in J2 host finding.

PPNs are highly damaging parasites with a tremendous impact on agriculture globally. *Meloidogyne* and *Globodera* spp. employ an extremely sophisticated and adaptive repertoire of effector molecules to subvert the plant host^64^. The complexity and diversity of these organisms mean that crop Resistance (R) genes are either unavailable, or are insufficiently durable, to protect crops over the long term^65^. Our reliance on synthetic chemical pesticides for their management can also have negative consequences to the environment and human health. Novel approaches, which are safe and effective, are required to control these parasites.

Preventing the act of PPN host-finding and invasion represents an attractive intervention strategy for crop protection, and one that could be developed through manipulation of crop root exudate composition. This strategy has been effectively demonstrated against parasitic plants within the *Striga* genus^17,27–30^, and could prevent secondary crop infection events. Our data demonstrate that crop plant gene expression can be modulated to alter exudate composition, as well as hatching and attraction of several important PPN species.

The GC-MS dataset reveals several compounds that are elevated in root exudates following ABC gene knockdown. When considering this relative to the behavioural response of PPN species to the exudates, we would hypothesise that elevated compounds function as repellents. Our behavioural data corroborate this for hexadecanoic acid, which is elevated following knockdown of *ABC-G33*, and pentadecane, which is elevated in exudates following knockdown of both *ABC-C6* and *ABC-G33*. Whilst our analysis of individual compounds has not been exhaustive, these observations do provide some confidence in our ability to predict exudate compound function using these approaches. It is clear however, that several of the compounds we selected for subsequent behavioural characterisation have no influence on PPN behaviour in these assays, and that complex interactions are likely involved. It should also be noted that root exudates will contain many additional compounds, which cannot be identified by GC-MS alone.

pH is known to influence the speciation of organic acids. On the basis of known pKa values, hexadecanoic acid, octadecanoic acid and tetradecanoic acid would be found mostly in the form of conjugate base (control *PDS* exudates were pH 6.94, and experimental exudates with inoculated compounds ranged between pH 6.60–7.12). Whilst we can’t conclusively determine if the changes in microbe or parasite attraction result from pH related changes on exudate speciation, the fact that different behavioural outcomes are observed in response to exudates that were inoculated with different organic acids (with similar pKa values), indicates that this is unlikely to be the only influencing factor. Certainly, more research is required to determine the relative impact of exudate compositional change, relative to pH impact on speciation. Our experimental design has assessed individual exudate compounds in the context of collected exudates, rather than isolated compounds, which provides additional assurance that speciation is appropriate for the exudate context, and our results are therefore biologically relevant.

It is increasingly apparent that rhizosphere microbes are vital for plant health, and it has been shown that microbial chemotaxis is an important factor in the early colonisation of plant roots, and plant protection^3^. We assessed the impact that root exudate compositional changes had on the positive chemotaxis of beneficial (*B. subtilis*^66,67^) and pathogenic (*A. tumefaciens*) rhizosphere microbes to the experimental exudates. Our results indicate that *A. tumefaciens* was attracted to root exudates irrespective of the compositional changes following knockdown of either *ABC-C6* or *ABC-G33*. However, *B. subtilis* was significantly less attracted to root exudates following knockdown of *ABC-G33*. These data indicate that approaches to rhizosphere engineering, through the manipulation of root exudate composition, will need to assess a wide range of relevant organismal interactions. In this study, we used the domesticated *B. subtilis* strain 168 as an experimental model for *B. subtilis* chemotaxis. *B. subtilis* 168 does not form biofilms, unlike the undomesticated parental strain *B. subtilis* NCIB 3610^68,69^. We found that *B. subtilis* NCIB 3610 would form biofilms within our chemotaxis assay timeframe, making it difficult to enumerate individual cells that were attracted between experimental treatments. However, there may be additional implications for plant-microbe interactions, particularly in terms of biofilm and *vir* gene induction.

Our study relies upon a new approach to VIGS inoculation, which uses topical application of *A. tumefaciens* to seedling leaves, in a suspension containing the non-ionic surfactant and wetting agent, Silwet-L77. Unlike blunt syringe inoculation, this approach does not trigger growth stunting effects in the treated plant and promotes longer lasting gene knockdown. In the context of this study, using a transient gene silencing approach, such as VIGS has several conceptual benefits relative to other approaches that constitutively inhibit target gene expression. Transient knockdown can limit downstream secondary impacts in plants relative to constitutively inhibited genes by CRISPR-Cas9 or transgenic RNAi approaches, which could lead to false positive phenotypes. This phenomenon could develop as a function of biochemical knock-on effects that manifest phenotypically, but do not relate exclusively to target gene function, as previously suggested^55^. Conceptually, we should have higher confidence in phenotypes recorded at earlier time points following loss of function analyses, which VIGS can facilitate. VIGS may also minimise genetic compensation, which occurs through transcriptional rescue of aberrant phenotypes, by counter-balanced expression of related genes. This can generate false negative phenotypes and is especially prominent in large gene families^70^. Rossi *et al*. indicate that genetic compensation can be avoided or reduced using transient knockdown^70^. VIGS typically results in a lower level of target gene knockdown than does transgenic dsRNA production and RNAi *in planta*^71^, which may also reduce genetic compensation processes. VIGS is extremely cost-effective relative to other functional genomics approaches, in terms of both reagents and personnel time. It also represents the highest throughput reverse genetics tool available across a number of crop plants, and can be used to rapidly probe parasite interactions^72^. VIGS can also be used to silence multiple genes simultaneously^73^.

Collectively, the data generated in this study support efforts to manipulate crop plant genes and promote beneficial rhizosphere interactions. This could occur through breeding, or biotechnology, and could support the sustainable intensification of global agriculture, through the rational and targeted exploitation of crop metabolic potential.

## Materials and Methods

### Virus induced gene silencing

*Solanum lycopersicum* (cv. Moneymaker) seedlings were sterilised by rinsing in 1% sodium hypochlorite (from a diluted commercial bleach) for no more than 1 minute. Seeds were then rinsed in sterile ddH_2_O three times for no more than 2 minutes per wash. ddH_2_O was removed and seeds were sown on 0.5x MS agar plates (half strength Murashige and Skoog (MS) basal salt mixture, 2 mM Morpholinoethanesulfonic acid (MES), 1.5% agar (w/v), pH 5.7). Seeds were stratified at 4 °C for 48 h in darkness before transfer to 23 °C with 16 h of white light (140–160 μE.m^−1^.s^−1^)/8 h. Seedlings were transferred to John Innes number 2 compost upon cotyledon emergence.

The tobacco rattle virus (TRV) VIGS vector, pTRV2, was modified to contain a 200 bp fragment of the tomato *PDS* gene (Solyc03g123760; pTRV2-*PDS*). We used a co-silencing system as previously described^73,74^ by generating pTRV2-*PDS-ABC-C6* and pTRV2-*PDS-ABC-G33* plasmids containing contiguous 200 bp fragments of the *PDS* gene sequence, followed by 200 bp of the gene of interest (either *ABC-C6* [Solyc08g006880] or *ABC-G33* [Solyc01g101070]). *Agrobacterium tumefaciens* strain GV3101 was used for VIGS throughout. *A. tumefaciens* cultures were transformed by electroporation, and stored as single use glycerol stocks. Briefly, 30 ng plasmid was added to 50 µl thawed electro-competent *A. tumefaciens* cells on ice, and then gently mixed by pipette. A single pulse of 2.2 kV was delivered to bacteria in a pre-chilled 1 mm gap cuvette. Cells were suspended in 1 ml LB broth, and incubated at 28 °C for two hours at 200 rpm, before plating 50 µl on LB agar plates with 50 µg/ml kanamycin and 50 µg/ml gentamycin. Colonies were screened for successful transformation by colony PCR using universal pTRV backbone primers (see Table 1). Plants were inoculated with pTRV1/pTRV2 on the third day after transfer, between 2–4 pm. From this point, plants were covered with a foil-lined propagator (to maintain humidity) for approximately 18 h at 18 °C; the lower temperature is necessary to promote VIGS efficacy.

**Table 1:** qRT-PCR primer sequences.

Gene	Tomato Gene ID	Primer Sequences (5′–3′)
EF-α	Solyc06g005060	F: TACTGGTGGTTTTGAAGCTG
		R: AACTTCCTTCACGATTTCATCATA
PDS	Solyc03g123760	F: GAAGGCGCTGTCTTATCAGG
		R: GCTTGCTTCCGACAACTTCT
ABC-C6	Solyc08g006880	F: ACACCCTGGTTATATCTGTTTC
		R: AAAGACCCAGCAAGTAGTTATAG
ABC-G33	Solyc01g101070	F: GCAATGAGGCCAATGTTAAG
		R: TTGAAGGTTGTCATGTTCAATG
pTRV1		F: GAGGGGAAACAAGCGGTACA R: TACCTCGTTCCCAAACAGCC
pTRV2		F: ACTCACGGGCTAACAGTGCT R: GACGTATCGGACCTCCACTC

### Preparation of *A. tumefaciens* cultures for tomato inoculation

For each construct, a single use glycerol stock was thawed and inoculated into 5 ml of LB broth containing 50 µg/ml kanamycin and 50 µg/ml gentamycin. pTRV1 was divided into two cultures (2 × 5 ml). Cultures were then incubated in darkness for 24–48 h at 28 °C, with orbital agitation at 180 rpm, to an OD_600_ of between 0.75–1. *A. tumefaciens* cultures were then diluted to a total volume of 50 ml containing 50 µg/ml kanamycin and 50 µg/ml gentamycin, 10 mM MES, and 20 µM acetosyringone. Cultures were then incubated for 24 h at 28 °C, with orbital agitation at 180 rpm, after which they were normalised to an OD_600_ of 1 in infiltration buffer (200 μM acetosyringone, 10 mM MES, 10 mM MgCl_2_, pH 5.7). Cultures were covered with foil and incubated for 3 h at room temperature. Immediately before topical application, a pTRV1 culture was mixed in a 1:1 v/v ratio with pTRV2, pTRV2-*PDS*, pTRV2-*PDS-ABC-C6*, or pTRV2-*PDS-ABC-G33*, as appropriate. Silwet-L77 was then added to a final concentration of 0.02%. No Silwet-L77 was added for the blunt syringe leaf infiltration method.

### Leaf infiltration and topical application methods

Prior to inoculation, the plants were watered to approximately 0.7 Field Capacity (FC). Leaf infiltration by blunt syringe was conducted as previously described, with a total volume of 0.1 ml injected per plant^75,76^. For topical application, 0.02% Silwett was added to the cultures immediately prior to application. An autoclaved paintbrush (size 10) was used to apply the culture across both abaxial and adaxial surfaces of the cotyledons, as well as the hypocotyl. Five strokes were administered to each seedling with a freshly inoculated paintbrush. Inoculated plants were maintained at 18 °C, and covered with a foil-lined propagator for approximately 18 h. The propagator lid was then removed, and routine maintenance resumed at 18 °C with a 16 h light, 8 h dark cycle, as before.

### Plant phenotype analysis

On the day of *A. tumefaciens* inoculation, and each subsequent day for six days, photographs were taken of five randomly selected plants in each treatment group to track growth of cotyledons. During this period cotyledon growth was found to be linear, after which growth begins to plateau and true leaves emerge. Thus the rate of cotyledon growth can be expressed as a function of y = mx + c. The rate of growth of each leaf was used in one-way ANOVA and Tukey’s HSD post-hoc tests to compare treatments. Plants were checked daily for leaf bleaching phenotypes, indicative of PDS co-silencing. The number of plants with unambiguous bleaching phenotypes were recorded daily and expressed as a percentage of the total number of plants inoculated.

### qRT-PCR analysis of gene transcript knockdown

Plants with bleaching phenotypes were removed from their pots and washed thoroughly under running water to remove soil. Three plants comprised one biological replicate in each treatment group at the times indicated. The plant tissue was wrapped in tin foil and flash frozen. Tissue was then crushed with a pestle and mortar into a fine powder. Frozen tissue was transferred to a 1.5 ml plastic microcentrifuge tube and total RNA was extracted using the Simply RNA Purification kit and Maxwell 16 extraction robot, following the manufacturer’s instructions (Promega). 10 µg total RNA was treated with Turbo DNase according to the manufacturer’s instructions (Ambion). 1 µg of purified RNA was subsequently reverse transcribed into cDNA using the High Capacity RNA-to-cDNA Kit as per manufacturer’s instructions (Applied Biosystems). A reverse transcription reagent (RTr) control, and a reverse transcription minus the reverse transcripase (RT-) control for a randomly selected sample per batch, were included.

cDNA, and controls, were diluted 1/4 using nuclease free water. 2.5 µl template was used for each qRT-PCR reaction in a total of 12 µl with 666 nM of each primer and 1x SensiFAST SYBR No-ROX mix as per manufacturer’s instructions (BIOLINE). Technical PCR reactions for each sample were performed in triplicate for each target using a Rotorgene Q thermal cycler with the following regime: [95 °C × 10 min, 45 × (95 °C × 20 s, 60 °C × 20 s, 72 °C × 25 s) 72 °C × 10 min]. PCR efficiencies of each amplicon, and the corresponding cT value, were calculated using the Rotorgene Q software. Relative quantification of each target amplicon was obtained by an augmented comparative Ct method^77^, relative to the reference gene EF-α (Solyc06g005060). Ratio-changes in transcript abundance were calculated relative to pTRV2-PDS treated plants. Data were analysed by one-way ANOVA and Tukey’s HSD post hoc test. Oligonucleotide sequences are listed in Table 1.

### Exudate collection

Plants were removed from their pots as described above, and cleared of soil by rinsing under running water. Five plants comprised one biological replicate. Plants were bunched together and their roots placed inside a 50 ml screw-top centrifuge tube (Corning), containing 10 ml ddH20. Plants were maintained under a standard 16 h light, 8 h dark regime at 18 °C for the duration of exudate collection. After 24 h, at around 1 pm, plants were removed from the tubes and the exudate was passed through a 0.22 µM filter to remove root border cells and residual soil. Soil control samples (approximately 1 g soil in 10 ml 0.22 µM filtered ddH_2_0) were processed in the same way. For chemosensory and hatching experiments, exudate was stored in centrifuge tubes at 4 °C in darkness until use. For GC-MS metabolomics, exudates were stored at −80 °C immediately after filtering.

### Non-targeted exudate metabolite profiling by GC-MS

Root exudates were freeze dried in batches and stored at −80 °C until all samples had been processed (n = 40; 10 biological replicates for each treatment group). Samples were extracted with GC-grade dichloromethane (1 mL) (Sigma–Aldrich, St. Louis, MO, USA), vortexed for 10 s, sonicated for 5 min, and centrifuged at 14,000 rpm for 5 min. The organic phase was dried over anhydrous Na_2_SO_4_, concentrated to 50 µL under a gentle stream of N2 and then analysed (1.0 µL) by GC-MS on a 7890 A gas chromatograph linked to a 5975 C mass selective detector (Agilent Technologies, Inc., Santa Clara, CA, USA). The GC was fitted with a HP5 MS low bleed capillary column (30 m × 0.25 mm i.d., 0.25 µm) (J&W, Folsom, CA, USA). Helium at a flow rate of 1.25 ml min-1 served as the carrier gas. The oven temperature was programmed from 35 to 285 °C with the initial temperature maintained for 5 min then 10 °C min-1 to 280 °C, held at this temperature for 20.4 min. The mass selective detector was maintained at ion source temperature of 230 °C and a quadrupole temperature of 180 °C. Electron impact (EI) mass spectra were obtained at the acceleration energy of 70 eV. Fragment ions were analyzed over 40–550 m/z mass range in the full scan mode. The filament delay time was set at 3.3 min. A HP Z220 SFF intel xeon workstation equipped with ChemStation B.02.02. acquisition software was used. The mass spectrum was generated for each peak using Chemstation integrator set as follows: initial threshold = 5, initial peak width = 0.1, initial area reject = 1 and shoulder detection = on. The compounds were identified by comparison of mass spectrometric data and retention times with those of authentic standards and reference spectra published by library–MS databases: National Institute of Standards and Technology (NIST) 05, 08, and 11. Exudate compounds were quantified, and fatty acids detected in the organic phase were confirmed by derivatization using their methyl esters and analysed by GC/MS.

### Nematode culture and maintenance

*Meloidogyne* nematode species were maintained on *S. lycopersicum* (cv. Moneymaker). Four-week old plants were infected with 1,000 J2s. Eggs were extracted eight weeks post infection by first gently rinsing the root tissue free of soil and placing the tissue in a 500 ml Duran bottle. Tissue was then rinsed in 50% bleach for no more than one minute, and then in water, pouring the liquid through nested 180 µM, 150 µM, and 38 µM sieves arranged from largest pore size to smallest on the bottom. For maximum recovery, plant material was then crushed by hand and rinsed in water through the sieves. Eggs collected in the bottom sieve were pelleted and re-suspended in saturated sucrose. 1 ml ddH_2_0 was added to the solution, and eggs were collected by flotation in the water layer by centrifugation at 2000 rpm for 2 minutes. Eggs were collected and placed in a fine mesh filter hatchery (pore size 38 µm) in 5 ml spring water (autoclaved and sterilised by 0.22 µm filter). 50 µL antibiotic/antimycotic solution (Sigma) was added and eggs were incubated at 23 °C. Freshly hatched J2s were collected every second or third day in hydrophobically lined microcentrifuge tubes for use in chemosensory and infection assays within 48 h of hatch.

*Globodera pallida* (pathotype PA2/3) cysts were reared on field grown potatoes (cv. Desiree). Two 10 week-old tomato (cv. Moneymaker) plants were grown under greenhouse conditions. 1 L ddH20 was poured into the soil and root stock of each pot, and collected after passing through the pot. Additional ddH20 was poured through the pots until the total collection volume reached 1 L. The collected exudate solution was passed through a 0.22 µM filter and stored in glass jars at 4 °C until use. For chemosensory assays and infection assays, *G. pallida* cysts were hatched in 1:1 (v/v) tomato exudate diluted in ddH20 and collected at two to three-day intervals in hydrophobically lined microcentrifuge tubes.

### Soil infection assays

Tomato plants exhibiting the co-silenced leaf bleaching phenotype were selected at around 21 days post inoculation, and then challenged with 250 J2s. All treatments were blinded from this stage. Six weeks after J2 inoculation, galls and cysts could be identified. These were counted for each experimentally infected plant.

### PPN chemosensory assays

Chemosensory assays were conducted as before^35,45^ by making a solid agar base (3 ml of 1.5% (w/v) agar) in a 60 mm Petri dish. 3 ml of smooth 0.5% (w/v) agar slurry was then poured on top of the solid base. Slurry was prepared by continuous mixing of liquid 0.5% agar until fully cooled. Approximately 150 J2s were then added to the centre of the arena under a microscope. Plates were covered and left in darkness at room temperature (20 °C) overnight on a vibration free bench (for approx. 16 h). The numbers of nematodes observed in the different regions of the arena were then counted under a light microscope. The Chemosensory Index (CI) of each assay was calculated using the formula CI = (A − B)/A + B, where A is the number of worms counted in the root exudate zone of the assay plate, and B is the number of worms counted in the water control zone of the assay plate^35,45^.

### PPN egg hatching assays

Tomato root exudate (collected as described above) was diluted in a 1:1 ratio with ddH_2_O. 500 µl of diluted exudate was dispensed into wells of a 24-well culture plate. To each exudate sample, between 15 and 20 *G. pallida* cysts were added. Four biological replicates were prepared for each treatment group. The spaces between wells were half filled with ddH20 and the plates wrapped in parafilm to reduce evaporation and changes of volume throughout the experiment. Plates were incubated at 17 °C for 21 days. Each well was checked daily for signs of nematode emergence. Following the first day of emergence, J2s were counted every 48 h. After approximately 3 weeks, the remaining unhatched eggs/J2s were counted in order to obtain a cumulative percentage hatch rate for each exudate sample. The number of emerging J2s at each time point was converted to a cumulative percentage, which was plotted against time as previously described^78^. 2000 freshly extracted *M. incognita* or *M. javanica* eggs were incubated in 500 µl of experimental plant root exudates. The ratio of unhatched to hatched J2s was then recorded every second or third day. These ratio values were converted into percentages and plotted relative to time, as previously described. A cumulative percentage hatch rate was defined for each sample. For each batch of eggs, triplicate counts of unhatched and hatched J2s were made. Four experimental replicates were used to calculate means. The area under the curve from the cumulative percentage hatch (AUC) was estimated by trapezoidal integration, as described by Campbell & Madden (1990).

### Microbe chemotaxis assays

Microbial chemotaxis assays were conducted broadly as in Allard-Massicotte *et al*.^3^. *B. subtilis* (168) and *A. tumefaciens* (AGL-1) were inoculated onto LB agar plates and spread to single colonies. One day-old colonies were inoculated into 3 ml LB broth and orbitally rotated at 180 rpm, overnight (28 °C for *A. tumefaciens* and 37 °C for *B. subtilis*). Cultures were pelleted at 8,000 rpm for 10 min, and the cells were washed in 1.5 ml chemotaxis buffer (10 mM Potassium Phosphate Buffer, pH 7.0), 0.1 mM EDTA, 0.05% glycerol, 5 mM Sodium-d, l-Lactate, 0.14 mM CaCl_2_, 0.3 mM (NH_4_)_2_SO_4_). Cells were collected by centrifugation and subsequently re-suspended in fresh chemotaxis buffer to an OD_600_ of 0.002. 200 μl of cell suspension was added to each well of a 96-well plate; ten replicates for each experimental group.

1 μl microcapillary tubes (Sigma-Alrdich) were filled with either: (i) experimental root exudates (*ABC-C6, ABC-G33*, or *PDS*), (ii) positive (1 mM malic acid), or negative (ddH_2_O) controls. Loaded microcapillary tubes were placed into the cell suspension wells of the 96-well plate for 1 h, and maintained at 23 °C. During this time, planktonic *B. subtilis* or *A. tumefaciens* cells could migrate towards, and into, the microcapillary tube. Following the 1 h assay timecourse, the capillary tubes were removed. Excess cell suspension was removed from the outside of each capillary tube by rinsing briefly with ddH_2_O. The 1 µl content of each capillary tube was ejected into 99 µl of chemotaxis buffer by positive pressure. 20 µl of each solution was spread onto a 1.5% LB agar plate. LB plates were sealed with parafilm, and incubated at 28 °C for *A. tumefaciens*, or 37 °C for *B. subtilis* for 48 h. Colony forming units were counted for each replicate plate.

## Supplementary information

Supplementary Dataset
